# Supplementary material for: Neuroprotective Effect of Curcumin-Loaded RGD Peptide-PEGylated Nanoliposomes
Source: Pharmaceutics. 2023 Nov 24;15(12):2665. doi: 10.3390/pharmaceutics15122665 (PMC10747044; doi:10.3390/pharmaceutics15122665)
Supplement: Supplementary file 1 [file pharmaceutics-15-02665-s001.zip › pharmaceutics-2660987-supplementary.pdf]

Supporting information

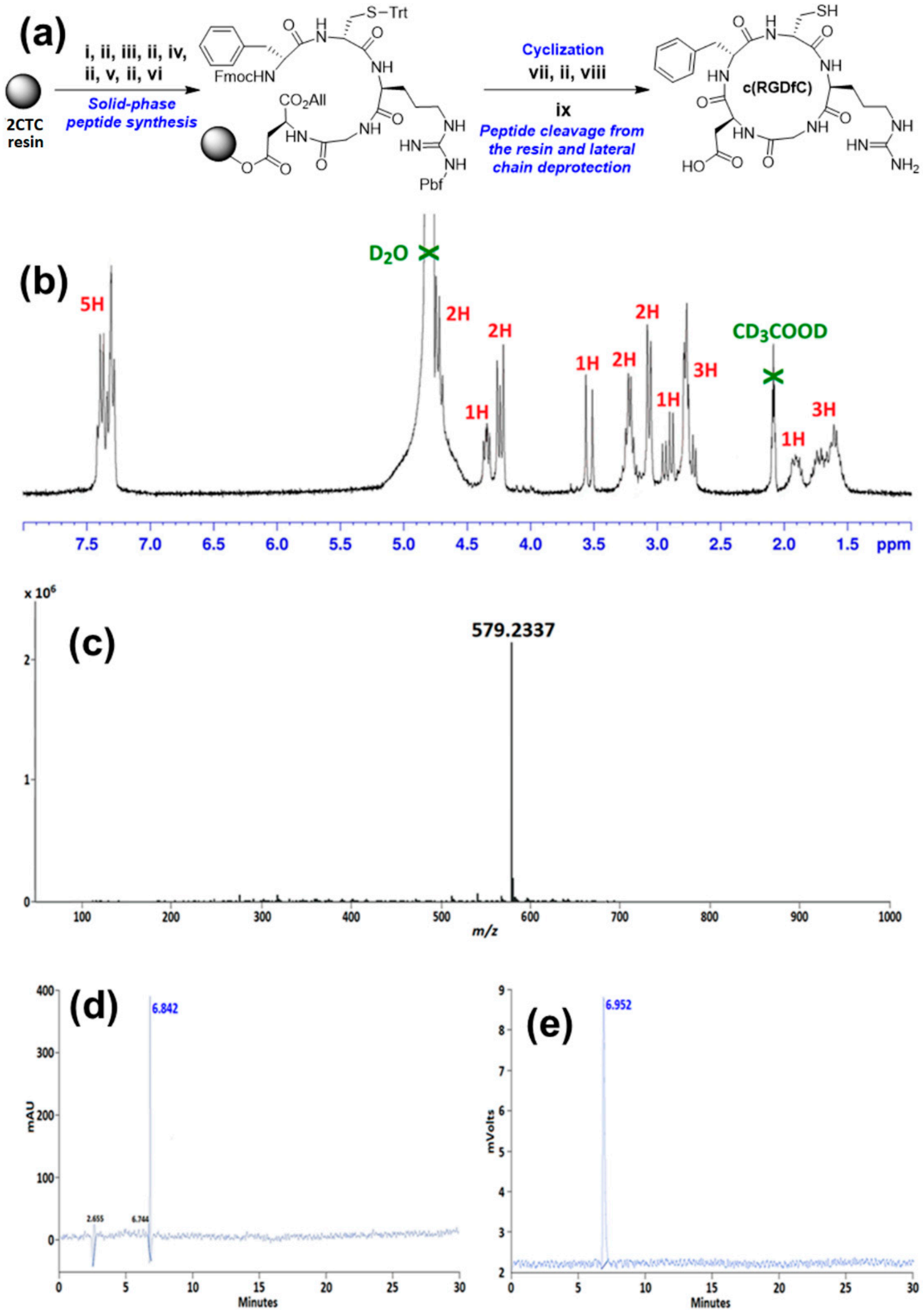

Figure S1. Synthesis and characterizations of c(RGDfC). (a) SPPS synthesis. (b) ^{1}H NMR spectrum (300 MHz) in D_2O/CD_3COOD (98/2, v/v). (c) HRMS spectrum. RP-HPLC chromatogram was detected using (d) UV-visible (λ_{abs} = 214 nm) and (e) fluorescence (λ_{abs} = 257 nm and λ_{em} = 274 nm).
